# Adverse events of a third dose of BNT162b2 mRNA COVID-19 vaccine among Korean healthcare workers

**DOI:** 10.1097/MD.0000000000033236

**Published:** 2023-03-17

**Authors:** Dong Yeop Lee, Dong Yoon Kang, Eunjung Kim, Se-joo Lee, Ji Hyeon Baek, Jin-Soo Lee, Mi Youn Park, Jae Hyoung Im

**Affiliations:** a Department of Preventive Medicine, Ulsan University Hospital, Ulsan, Republic of Korea; b Infection Control Unit, Inha University Hospital, Incheon, Korea; c Division of Infectious Diseases, Department of Internal Medicine, Inha University College of Medicine, Incheon, Republic of Korea; d Department of Nursing, Inha University College of Medicine, Incheon, Republic of Korea.

**Keywords:** adverse event, COVID-19, injection site reaction, mRNA vaccine, vaccine

## Abstract

Due to the urgency of controlling the coronavirus disease 2019 pandemic, coronavirus disease 2019 messenger ribonucleic acid (mRNA) vaccines have been expeditiously approved and introduced in several countries without sufficient evaluation for adverse events. We analyzed adverse events among Korean healthcare workers who received all 3 doses of the BNT162b2 mRNA vaccine. This survey was conducted among hospital workers of Inha University Hospital who had received the BNT162b2 mRNA vaccine for their first, second, third rounds, and using a diary card. The surveyed adverse events included local (redness, edema, and injection site pain) and systemic (fever, fatigue, headache, chill, myalgia, arthralgia, vomiting, diarrhea, pruritis, and urticaria) side effects and were divided into 5 grades (Grade 0 = none – Grade 4 = critical). Based on adverse events reported at least once after any of the 3 doses, the most common systemic adverse reactions were chills and headache (respectively, 62.6%, 62.4%), followed by myalgia (55.3%), arthralgia (53.4%), fatigue (51.6%), pruritus (38.1%), and fever (36.5%). The frequency and duration of adverse events were significantly greater in women (*P* < .05) than men. Except for redness, pruritus, urticaria, and most adverse reactions had a higher rate of occurrence after the third dose in subjects who also had reactions with the second dose. However, grade 4 adverse events did occur with the third dose in some patients, even if there were no side effects with the first and second doses. Adverse events experienced with the first and second doses of the BNT162b2 mRNA vaccine in Korean healthcare workers increased the incidence of adverse events at the time of the third dose. On the other hand, grade 4 adverse events could still occur with the third dose even though there were no side effects with the first and second doses.

## 1. Introduction

First reported in November 2019, coronavirus disease 2019 (COVID-19) has developed into a pandemic, causing millions of deaths worldwide.^[1]^ Vaccination has been proposed as an important intervention to end the pandemic, various types of vaccines are currently in use or under development.^[2]^ Although several vaccines, including adenovirus vector-based, messenger ribonucleic acid (mRNA)-based, nonreplicating viral vector, recombinant protein, and inactivated vaccines, have been authorized for use in countries worldwide, mRNA vaccines are the predominant type used at this time.^[3–7]^

Although the efficacy of mRNA vaccines in preventing infection by severe acute respiratory syndrome coronavirus 2 (SARS-CoV-2) wild-type is inferior to that of SARS-CoV-2 variants, mRNA vaccines have been demonstrated to be effective in preventing progression to severe disease.^[8,9]^ In addition, an updated bivalent mRNA vaccine is being prepared, so it is expected that the mRNA COVID-19 vaccine will continue to play an important role for older adults and immunocompromised patients in the immediate future.^[10]^

For mRNA vaccines against COVID-19, mRNA encoding the SARS-CoV-2 spike protein is injected into the patient. The injected mRNA uses ribosomes to synthesize the viral spike protein that stimulates immunity.^[11]^ Although the development of mRNA vaccines started a decade ago, the mRNA SARS-CoV-2 vaccines were approved and progressed at an unprecedented rate due to the urgency of controlling the COVID-19 pandemic.^[12,13]^ Therefore, long-term adverse side effects of mRNA vaccines and side effects during repeated vaccination were not observed for a sufficient period. In addition, as the vaccine was released with limited data, it is necessary to supplement the data. We analyzed the adverse events associated with the first, second, and third doses of the BNT162b2 mRNA vaccine in Korean healthcare workers.

## 2. Methods

### 2.1. Participants and vaccination schedule

Healthcare workers in a tertiary hospital (Incheon, Republic of Korea) received 3 doses of the BNT162b2 mRNA COVID-19 vaccine. Vaccination was administered in 2021 between March 9 and March 12 (first dose), March 30 and April 2 (second dose, 3 weeks apart), and October 25 to November 29 (third dose, 6–7 months apart).

### 2.2. Surveillance for adverse events

Paper diary cards were used to survey adverse events of the BNT162b2 mRNA COVID-19 vaccine. Among the submitted diary cards, only those who responded to all adverse events for at least 1 day in all surveyed items were considered valid.

### 2.3. Criteria for adverse events

The surveyed items of adverse events, which included local (redness, edema, and pain) and systemic reactions (fever, fatigue, headache, chills, myalgia, arthralgia, nausea, and diarrhea), were divided into 5 grades (0 = none to 4 = critical). A description of what constituted each severity grade for each adverse event is listed in Table 1.

**Table 1:** Operational definition.

Adverse effects	Grade				
	0	1	2	3	4
Injection site pain	No pain	No disruption to daily life	A little interruption in daily life	Severe disruption of daily life	Skin necrosis
Edema	No swelling or < 2cm	2–5 cm in diameter	5–10cm in diameter	More than 10 cm in diameter	Skin necrosis
Redness	No swelling or < 2 cm	2–5 cm in diameter	5–10cm in diameter	More than 10 cm in diameter	Skin necrosis
Fatigue	No fatigue	No disruption to daily life	A little interruption in daily life	Severe disruption of daily life	Emergency room visits or hospital admissions
Myalgia	No myalgia	No disruption to daily life	A little interruption in daily life	Severe disruption of daily life	Emergency room visits or hospital admissions
Headache	No headache	No disruption to daily life	A little interruption in daily life	Severe disruption of daily life	Emergency room visits or hospital admissions
Chills	No chills	No disruption to daily life	A little interruption in daily life	Severe disruption of daily life	Emergency room visits or hospital admissions
Arthralgia	No arthralgia	No disruption to daily life	A little interruption in daily life	Severe disruption of daily life	Emergency room visits or hospital admissions
Pruritus	No pruritus	No disruption to daily life	A little interruption in daily life	Severe disruption of daily life	Emergency room visits or hospital admissions
Vomiting	No vomiting	No disruption to daily life	A little interruption in daily life	Severe disruption of daily life	Emergency room visits or hospital admissions
Fever	36.0°C–37.9°C	38.0°C–38.4°C	38.5°C–38.9°C	39.0°C–39.9°C	More than 40.0°C
Diarrhea	No diarrhea	No disruption to daily life	A little interruption in daily life	Severe disruption of daily life	Emergency room visits or hospital admissions
Urticaria	Usual state	< 20	20–49	More than 50	Emergency room visits or hospital admissions

### 2.4. Data analysis

Independent sample *t* test and 1-way ANOVA were performed to compare the difference in duration of adverse effects by gender and age. Multiple regression analysis was used to analyze the correlation between age and duration of adverse events. Logistic regression analysis was used to analyze the effect of adverse effects occurring after the first and second doses on the adverse effects occurring after the second and third doses. The analysis was performed by correcting for adverse effects occurring after the first and second doses, as well as general characteristics, such as gender and age. Data analysis was performed using SPSS statistical software, version 26.0 (SPSS, Inc., Chicago, IL).

### 2.5. Ethics statement

The requirement of obtaining informed consent was waived by the Inha University Institutional Review Board.

## 3. Results

### 3.1. General characteristics

**Figure 1.**
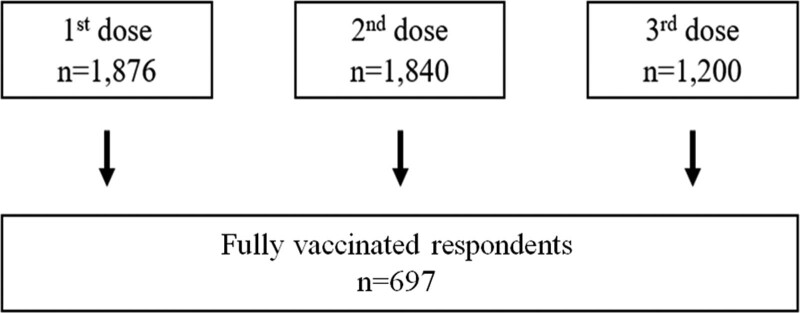
Flow of the present study. Among the 2498 vaccinated workers, a survey was conducted targeting 1876 people who received the 1st dose, 1840 people who received the 2nd dose, and 1200 people who received the 3rd dose. Among them, the data of 697 people who took the same survey for the 1st, 2nd and 3rd vaccinations were analyzed.

A total of 2498 workers received the first, second, and third doses of the Pfizer BNT162b2 mRNA vaccine. Among them, 56 workers were not vaccinated with a second or third dose. The reasons for not having a second or third dose were as follows: on leave of absence (n = 7), experienced severe side effects (n = 9), diagnosed with COVID-19 within 3 months from the second or third dose (n = 4), misinformed that COVID-19 vaccination is contraindicated during pregnancy (n = 13), and disagreed with vaccination (n = 23). Of the 2442 workers who received all 3 doses, 1876 workers responded to the questionnaire after the first dose, 1840 workers after the second dose, and 1200 workers after the third dose. Among these respondents, 697 workers answered all the questionnaires after each of the 3 doses and had their data analyzed (Fig. 1). Of the 697 workers analyzed, 586 (84.07%) were female. The age distribution of the sample population was as follows: 20 to 29 years old (n = 248), 30 to 39 years old (n = 142), 40 to 49 years old (n = 174), 50 to 59 years old (n = 116), 60 + years old (n = 17) (Table S1, Supplemental Digital Content, http://links.lww.com/MD/I648).

### 3.2. Frequency and duration of adverse events

**Figure 2.**
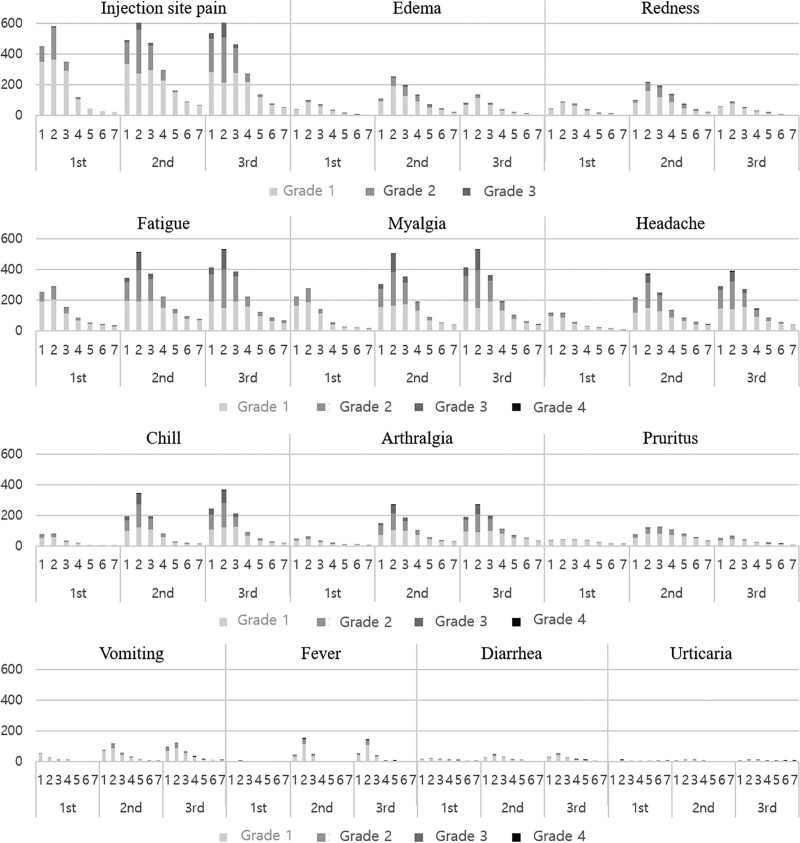
Severity of adverse events for 7 days after 1st, 2nd, and 3rd dose of BNT162b2 mRNA COVID-19 vaccine. Figure 2. shows the change by date of each adverse event in 697 respondents. With most adverse events, the 2nd day was when the most adverse events were reported. The reports of adverse events decreased thereafter. COVID-19 = coronavirus disease 2019, mRNA = messenger ribonucleic acid.

**Figure 3.**
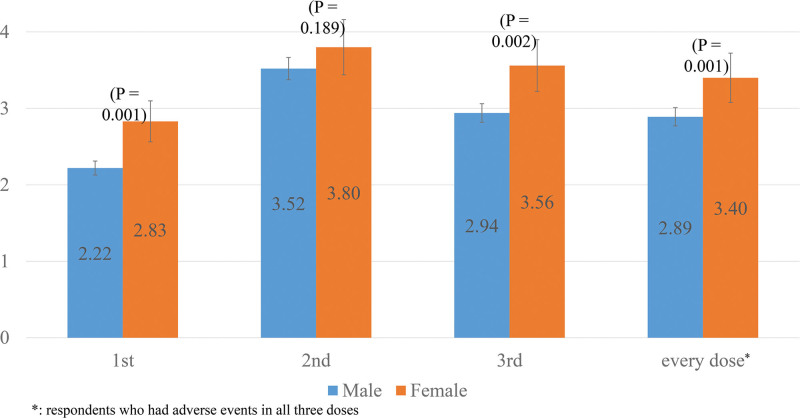
Comparison of duration of adverse events between sexes. Figure 3. shows the duration of adverse events by sex. The frequency of side effects reported in women was statistically significantly higher after each dose.

The most common local adverse event was injection site pain, which occurred after at least one dose in 684 workers (98.0%). Among the systemic adverse reactions, headache and chills (respectively, 62.4%, 62.6%) was the most common, followed by myalgia (55.3%), arthralgia (53.4%), fatigue (51.4%), pruritus (38.2%), fever (36.5%), vomiting (36.3%), and diarrhea (19.5%) (Fig. 2 and Figure S1, Supplemental Digital Content, http://links.lww.com/MD/I649). Urticaria had the lowest frequency of adverse events (6.3%) among those reported. The duration of adverse events was significantly longer in females with each vaccination dose (*P *< .05) (Fig. 3). Subjects between the ages 30 to 39 and 40 to 49 had more frequent side effects and longer duration than those aged 20 to 29 and 50 to 59.

### 3.3. Effects of first and second dose on adverse events occurring after the second and third dose

For most of the adverse events, except for fever and injection site redness, workers who experienced an adverse event after the first dose had a higher probability of experiencing an adverse event after the second dose. If any events other than urticaria, injection site redness, and pruritus occurred after the second dose, the probability of experiencing an adverse event increased after the third dose. Experiencing redness at the inoculation site after the first and second dose had a low correlation with its occurrence after the second and third dose. As for pruritis and urticaria, the experience of these adverse events after the first dose was correlated with the second dose, but not after the second dose with the third dose (Table 2).

**Table 2:** Effect of 1st and 2nd doses on adverse events occurring during 2nd and 3rd doses.

Adverse events	1st → 2^nd^ vaccination		2nd → 3^rd^ vaccination	
	OR (95% CI)	*P* value	OR (95% CI)	*P* value
Injection site pain	6.75 (3.52–12.97)	< .01	4.02 (1.93–8.40)	< .01
Injection site swelling	3.80 (2.01–7.16)	< .01	4.95 (2.78–8.81)	< .01
Injection site redness	0.79 (0.42–1.50)	.47	1.39 (.73–2.64)	.32
Myalgia	2.19 (1.41–3.38)	< .01	3.16 (1.87–5.35)	< .01
Fatigue	2.26 (1.44–3.53)	< .01	1.83 (1.10–3.04)	.02
Headache	2.44 (1.57–3.78)	< .01	2.83 (1.92–4.18)	< .01
Chills	2.08 (1.26–3.42)	< .01	3.13 (2.13–4.61)	< .01
Arthralgia	2.16 (1.26–3.72)	< .01	3.35 (2.29–4.92)	< .01
Pruritus	2.22 (1.30–3.79)	< .01	1.36 (.78–2.37)	.28
Vomiting	2.34 (1.36–4.03)	< .01	2.96 (1.91–4.59)	< .01
Fever	2.14 (0.40–11.43)	.37	3.80 (2.48–5.82)	< .01
Diarrhea	3.19 (1.47–6.96)	< .01	4.76 (2.51–9.03)	< .01
Urticaria	33.91 (5.94–193.55)	< .01	<0.01 (<0.01)	.99
Adjusting value: Other adverse effect, Sex, Age				

CI = confidence interval, OR = odds ratio.

Table shows the effect of experiencing adverse events with the previous dose on adverse events at the next dose.

### 3.4. Severe adverse events

Adverse events rated at grade 3 to grade 4 were reported 4.4% of the time after the first dose, 31.5% after the second dose, and 33.8% after the third dose. The most common grade 4 adverse events reported were fatigue, myalgia, headache, and chills (Table S2, Supplemental Digital Content, http://links.lww.com/MD/I650). Nine patients reported grade 4 adverse events after the third dose. None of these patients showed milder adverse events with the second dose than the first dose. One patient experienced a grade 4 fever after the third dose even with no fever after both the first and second doses. The severity of adverse events was higher in patients aged 30 to 39 and 40 to 49 compared to those aged 50 to 59 (Table S3, Supplemental Digital Content, http://links.lww.com/MD/I651).

## 4. Discussion

In this study, adverse events after the first, second, and third dose were analyzed from 697 healthcare workers. In Korea, data are generally limited for adverse events up to the second dose of Pfizer COVID-19 vaccine. Data on adverse events from the third dose may be helpful in predicting data for subsequent fourth or annual vaccinations. In contrast to previous studies, this study analyzed responses from participants who answered all 3 questions, thereby allowing for a linear analysis of changes in the frequency and intensity of adverse reactions in each individual. This is a significant advantage over previous studies. In particular, considering the recent decrease in the use of the adenovirus vector COVID-19 vaccine, this study will be helpful in predicting adverse events in Koreans who will be vaccinated with the mRNA-type COVID-19 vaccine for several years to come.

In the large-scale randomized controlled trial of adverse events after the third dose of BNT162b, injection site pain was the most common adverse event, in agreement with our study.^[14]^ However, the incidence of this event in the previous study was 12.9%, much lower than the 90.24% in our study (based on third dose). In addition, the incidence of other adverse events, such as fatigue at 7.2%, chills at 4.6%, and fever at 4.8%, was also significantly different from those of 93.0%, 72.7%, and 36.9% in our study. Differences in age distribution, reporting methods, single healthcare worker group, and race/culture can be considered as possible causes. There have been a few studies on the differences in the incidence of adverse events between women and men. In the previous studies, the frequency of adverse events was higher in women than men, similar with our study.^[15,16]^

Another study showed no significant difference in the frequency of occurrence of adverse events after the second dose and third dose in physiological indicators using a smartwatch in 1609 people. In the study, however, few people received all 3 doses with BNT162b2. There was also no analysis of predictors of adverse events after the third dose in the study.^[17]^ In our study, adverse events after the first dose were associated with the occurrence of adverse events after the second dose, and adverse events after the second dose were also related to those after the third dose. Fever after the first dose was not significantly associated with side effects after the second dose, which is thought to be due to the low incidence (1%) of fever after the first dose. Considering that fever after the second dose was associated with fever after the third dose, it is thought that fever after a previous dose is helpful in predicting fever after the next dose. Conversely, redness, urticaria, and pruritis were not related, so it was thought that experiencing these with previous doses would not be helpful in predicting those following future doses.

It is interesting that there were no grade 4 adverse events after the third dose in respondents who reported reduced side effects after the second dose compared to the first dose. Since patients who experienced severe adverse events after the first and second doses (N = 9) were excluded from receiving the third dose, it is possible that this may have influenced the previously stated finding. In addition, there was a patient who had a grade 4 fever with the third dose who did not have fever after both the first and second doses, so caution is needed in interpreting these findings.

Although not the main purpose of this study, it was noteworthy that there were 13 cases of pregnancy among the reasons for not receiving the third dose. The COVID-19 vaccine has been shown to be relatively safe in pregnant women. Pregnancy is a high-risk group for COVID-19, and a vaccine is an important preventative measure because antiviral use is limited. Nevertheless, public awareness is still insufficient, and there is a need to further publicize the need and safety of vaccines in pregnant women.^[18]^

This study has some limitations. First, the number of responders is relatively small. Second, it is limited to healthcare workers, so it cannot represent the whole Korean population. Third, the effect of antipyretic drugs was not excluded. Fourth, the response rate was lower after the third dose than after the first and second doses, so bias may have occurred. Fifth, the majority of Grade 4 adverse events in our study were met by the subject’s presentation to the emergency room. As all the subjects worked in a hospital, access to the emergency room was higher than that of nonmedical personnel. Therefore, the possibility that Grade 4 adverse events was overexpressed cannot be excluded.

The most common local adverse event in this study was injection site pain, and the most common systemic adverse event was headache and chills. Excluding redness, urticaria, pruritis, and the experience of adverse events after the second dose increased the probability of adverse events after the third dose.

## Author contributions

**Conceptualization:** Dong Yoon Kang, Jae Hyoung Im.

**Formal analysis:** Dong Yeop Lee.

**Data curation:** Eunjung Kim, Se-joo Lee, Ji Hyeon Baek, Jin-Soo Lee, Mi Youn Park.

**Writing – original draft:** Dong Yeop Lee.

**Writing – review & editing:** Dong Yoon Kang, Jae Hyoung Im.

## Supplementary Material

## References

[1] Zhu N Zhang D Wang W. A novel coronavirus from patients with pneumonia in China, 2019. N Engl J Med. 2020;382:727–33. 31978945 10.1056/NEJMoa2001017 PMC7092803

[2] Tregoning JS Brown E Cheeseman H. Vaccines for COVID-19. Clin Experimental Immunol. 2020;202:162–92. 10.1111/cei.13517 PMC7597597 32935331

[3] Kashte S Gulbake A Sf EI. COVID-19 vaccines: rapid development, implications, challenges and future prospects. Hum Cell. 2021;34:711–33. 33677814 10.1007/s13577-021-00512-4 PMC7937046

[4] Knoll MD Wonodi C. Oxford–AstraZeneca COVID-19 vaccine efficacy. Lancet. 2021;397:72–4. 33306990 10.1016/S0140-6736(20)32623-4 PMC7832220

[5] Skowronski DM De Serres G. Safety and efficacy of the BNT162b2 mRNA Covid-19 vaccine. N Engl J Med. 2021;384:1576–7. 33596348 10.1056/NEJMc2036242

[6] Mahase E. Covid-19: Moderna vaccine is nearly 95% effective, trial involving high risk and elderly people shows. BMJ. 2020;371.

[7] Heath PT Galiza EP Baxter DN. Safety and efficacy of NVX-CoV2373 Covid-19 vaccine. N Engl J Med. 2021;385:1172–83. 34192426 10.1056/NEJMoa2107659 PMC8262625

[8] Andrews N Stowe J Kirsebom F. Covid-19 vaccine effectiveness against the Omicron (B. 1.1. 529) variant. N Engl J Med. 2022;386:1532–46. 35249272 10.1056/NEJMoa2119451 PMC8908811

[9] Burki TK. Omicron variant and booster COVID-19 vaccines. Lancet Respir Med. 2022;10:e17. 34929158 10.1016/S2213-2600(21)00559-2 PMC8683118

[10] Chalkias S Harper C Vrbicky K. A bivalent Omicron-containing booster vaccine against Covid-19. N Engl J Med. 2022;387:1279–91. 36112399 10.1056/NEJMoa2208343 PMC9511634

[11] Huang Q Zeng J Yan J. COVID-19 mRNA vaccines. J Genet Genomics. 2021;48:107–14. 34006471 10.1016/j.jgg.2021.02.006 PMC7959685

[12] Deering RP Kommareddy S Ulmer JB. Nucleic acid vaccines: prospects for non-viral delivery of mRNA vaccines. Expert Opin Drug Deliv. 2014;11:885–99. 24665982 10.1517/17425247.2014.901308

[13] Koritala T Hussain A Pleshkova Y. A narrative review of emergency use authorization versus full FDA approval and its effect on COVID-19 vaccination hesitancy. Infez Med. 2021;29:339–44. 35146338 10.53854/liim-2903-4 PMC8805497

[14] Moreira Jr ED Kitchin N Xu X. Safety and efficacy of a third dose of BNT162b2 COVID-19 vaccine. New England J Med. 2022;386:1910–1921. 35320659 10.1056/NEJMoa2200674 PMC9006787

[15] El-Shitany NA Bagher AM Binmahfouz LS. The adverse reactions of Pfizer BioNTech COVID-19 vaccine booster dose are mild and similar to the second dose responses: a retrospective cross-sectional study. Int J Gen Med. 2022;15:6821–36. 36051568 10.2147/IJGM.S376316 PMC9426871

[16] Green MS Peer V Magid A. Gender differences in adverse events following the Pfizer-BioNTech COVID-19 vaccine. Vaccines (Basel). 2022;10:233. 35214694 10.3390/vaccines10020233 PMC8875740

[17] Mofaz M Yechezkel M Guan G. Self-reported and physiologic reactions to third BNT162b2 mRNA COVID-19 (Booster) vaccine dose. Emerg Infect Dis. 2022;28:1375–83. 35654410 10.3201/eid2807.212330 PMC9239876

[18] Moro PL Olson CK Zhang B. Safety of booster doses of Coronavirus Disease 2019 (COVID-19) vaccine in pregnancy in the vaccine adverse event reporting system. Obstet Gynecol. 2022;140:421–7. 35926203 10.1097/AOG.0000000000004889 PMC9377365

