# Supplementary material for: Adverse events of a third dose of BNT162b2 mRNA COVID-19 vaccine among Korean healthcare workers
Source: Medicine (Baltimore). 2023 Mar 17;102(11):e33236. doi: 10.1097/MD.0000000000033236 (PMC10018524; doi:10.1097/MD.0000000000033236)
Supplement: Supplementary file 1 [file medi-102-e33236-s001.pdf]

Supplementary table 1. General characteristics

Age (23 – 65, 37.74±11.26)	Sex	
	Male	Female
20s	22	226
30s	25	117
40s	29	145
50s	33	83
60s	2	15
