# Supplementary figures and images for: Adverse events of a third dose of BNT162b2 mRNA COVID-19 vaccine among Korean healthcare workers
Source: Medicine (Baltimore). 2023 Mar 17;102(11):e33236. doi: 10.1097/MD.0000000000033236 (PMC10018524; doi:10.1097/MD.0000000000033236)

Supplementary Figure 1. Frequency of adverse events after BNT162b2 mRNA COVID-19 vaccine

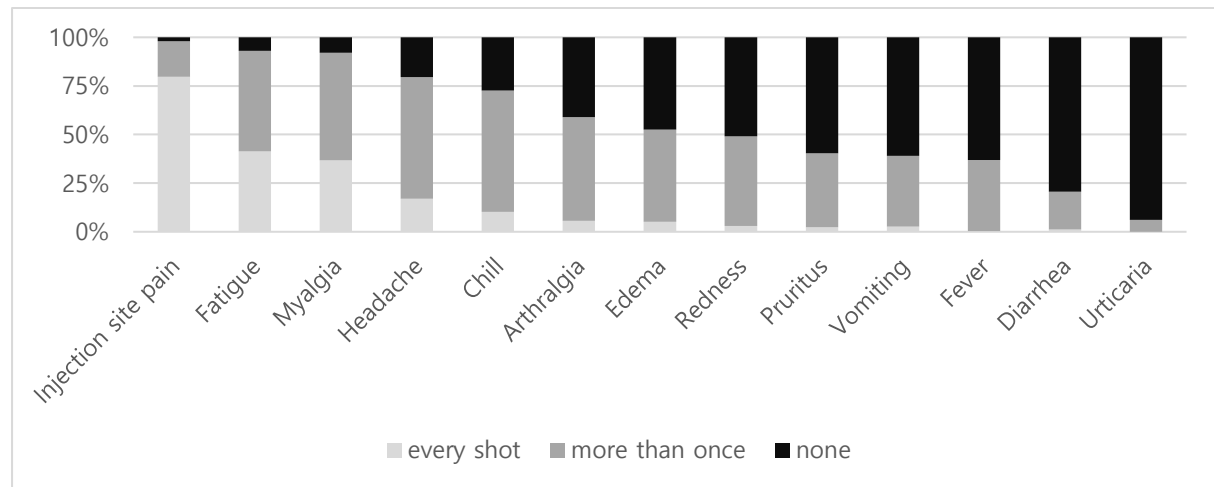

Supplement: Supplementary file 2 [file medi-102-e33236-s002.pdf]
