# Supplementary material for: Adverse events of a third dose of BNT162b2 mRNA COVID-19 vaccine among Korean healthcare workers
Source: Medicine (Baltimore). 2023 Mar 17;102(11):e33236. doi: 10.1097/MD.0000000000033236 (PMC10018524; doi:10.1097/MD.0000000000033236)
Supplement: Supplementary file 3 [file medi-102-e33236-s003.pdf]

Supplementary table 2. Grade 4 adverse events

Adverse events	1st dose (n=1)	2nd dose (n=4)	3rd dose (n=7)
Fever	0	1	2
Fatigue	0	2	2
Headache	0	2	2
Chills	0	2	2
Vomiting	0	1	2
Diarrhea	0	0	1
Myalgia	0	2	2
Arthralgia	0	1	1
Pruritus	0	0	1
Urticaria	1	0	0

*Subjects can make multiple answers in the questionnaire.
