# Supplementary material for: Adverse events of a third dose of BNT162b2 mRNA COVID-19 vaccine among Korean healthcare workers
Source: Medicine (Baltimore). 2023 Mar 17;102(11):e33236. doi: 10.1097/MD.0000000000033236 (PMC10018524; doi:10.1097/MD.0000000000033236)
Supplement: Supplementary file 4 [file medi-102-e33236-s004.pdf]

Supplementary table 3. Differences in severity according to age

Age	Average severity	
	M±SD	F(p)
20’	0.56±0.31[ab]	7.261 (<0.001)
30’	0.65±0.32[b]	
40’	0.65±0.34[b]	
≥50	0.51±0.25[a]	

Post hoc analysis: a<b
